# Improving postnatal social support using antenatal group-based psychoeducation: a cluster randomized controlled trial

**DOI:** 10.3389/fgwh.2025.1510725

**Published:** 2025-03-28

**Authors:** Marta Tessema, Muluemebet Abera, Zewdie Birhanu

**Affiliations:** ^1^ School of Midwifery, Institute of Health, Jimma University, Jimma, Ethiopia; ^2^ Department of Population and Family Health, Faculty of Public Health, Jimma, Ethiopia; ^3^ Department of Health, Behavior and Society, Faculty of Public Health, Jimma, Ethiopia

**Keywords:** social support, psychosocial support systems, postnatal care, randomized controlled trial, primary health care, maternal-child health services, depression, postpartum

## Abstract

**Background:**

Inadequate social support is the predominant cause of postnatal depression, which needs to be promoted through interventions. The objective of this study was to investigate the effect of antenatal group-based psychoeducation on improving postnatal social support.

**Methods:**

The trial design was a cluster randomized controlled trial. The study was conducted on 32 non-adjusted health centers (clusters) among 550 pregnant women. Using simple randomization, health centers were randomized into 16 intervention and 16 control groups. The intervention group received both standard prenatal care and group-based psychoeducation sessions, whereas the control group received standard prenatal care alone. The study included all pregnant women who were between 12 and 20 weeks gestation and had a Patient Health Questionnaire-9 <10 level of depression. We used a functional social support questionnaire in a face-to-face interview to assess social support at 12–20 weeks of gestation and 6 weeks postpartum. An intention-to-treat analysis was done. We used relative risk and a mixed-effects multilevel logistic regression for data analysis.

**Result:**

Out of 550 enrolled pregnant women, end-line data were collected from 511 participants, with an overall end-line response rate of 92.9%. Statistical analysis revealed that the intervention resulted in a substantial difference in all dimensions of social support between arms, although no difference was detected at baseline. As compared to that in controls, the total postnatal social support in the intervention clusters was considerably higher [190 (66.4%) vs. 88 (33.3%)], *P* = 0.001). Mothers who were under the intervention arms and received antenatal group-based psychoeducation were 2.04 times more likely to have postnatal social support (RR = 2.044, 95% CI: 1.684–2.481) compared to those who were under the control arms and received the usual care. Finally, mixed-effect analysis indicates that after adjusting for individual and community-level variables, the final model shows the intervention increased the total social support by 3.61 (AOR = 3.61, 95% CI: 2.14–6.09).

**Conclusion:**

The implementation of antenatal group-based psychoeducation intervention resulted in a statistically significant effect in improving postnatal social support. This intervention approach must therefore be implemented and promoted in maternal healthcare services.

**Clinical Trial Registration:**

https://pactr.samrc.ac.za/, identifier (PACTR 202203616584913).

## Introduction

Social support represents a person's sense of security and belongingness concerning a loving relationship, family, or community (1). Four behavioral dimensions are commonly used to categorize social support, namely, information, instrumental, emotional, and appraisal support (2). Researchers have extensively examined social support with individuals’ physical and emotional well-being (3). Similarly, studies indicate that perinatal women with a strong social support network manage stress better and are generally healthier than those without such a network (4, 5). It is used as an instrument in buffering and protecting against postpartum depression (PPD) by dampening increases in psychiatric vulnerability and life stressors (6). However, currently, an umbrella review study shows that perinatal depression affects up to 65% (7) of women globally, with pooled prevalence in low- and middle-income nations at 34% and 22.7%, respectively (8). In Ethiopia, the pooled prevalence of PPD is 21.9% (9). Correspondingly, the abovementioned studies indicate that for this high prevalence of PPD, the predominant risk factor is having poor social support (7–9). Furthermore, evidence shows that lack of social support and low intimate partner support during pregnancy have increased the chance of developing persistent postpartum depression more than sevenfold (10) and can compromise both the mother's and infant's well-being (11, 12).

Additionally, research has demonstrated an association between mothers’ social support demands and an increased risk of perinatal depression throughout the perinatal period. Perinatal depression rises throughout the three trimesters and during the early postpartum period (6, 8, 13, 14). In a similar vein, the demands of maternal social support and biological risk factors associated with postpartum depression, including the impact of placental corticotropic hormones, increase after mid-gestation, peaking during the third trimester and early postpartum period (6, 8, 13, 14). Moreover, postpartum depression is commonly preceded by antenatal depression, which has been associated with unfavorable obstetric outcomes and impaired child neurodevelopment (15–17), early cessation of breastfeeding (17–20), and receiving fewer preventive health services like vaccinations (21, 22). The WHO indicates that treating the depression of mothers leads to improved growth and development in the newborn and reduces the likelihood of diarrhea and malnutrition in children (23).

Therefore, as social support is an instrument in buffering and protecting against postpartum depression (PPD) by dampening increases in psychiatric vulnerability and life stressors, mothers should be aware of and prepare for social support during the early antenatal period, especially before mid-pregnancy when depression is less likely (6, 13, 14, 24). Because depressed mothers mostly face feelings of being overly anxious and irritable, avoid social contact, and develop perceived stigma (25), it is difficult for mothers to prepare a social support once they are depressed. Correspondingly, the WHO has recommended social support from the early prenatal period (26), but in most low- and middle-income countries, the provision of such a type of intervention is not adequately practiced during this period, as the focus has been mainly limited to pregnancy and childbirth education in relation to the physical health of the mother (27).

In Ethiopia, this limited support is given in the form of a birth preparedness and complication readiness plan, which primarily emphasizes instrumental support but is insufficient to cover all dimensions of support that are crucial for enhancing the mother's emotional well-being. Therefore, this study tries to improve all dimensions of social support in postnatal women through early antenatal group-based psychoeducation interventions.

Psychoeducation emphasizes imparting knowledge regarding the multifaceted nature of PPD and its impact on postnatal mothers, their infants, and their families together. The idea is that the more informed the care recipients and informal careers are about the effect of poor social support, the greater assistance the mother receives (28). Moreover, the intervention was administered in groups, which provided distinct advantages for the women. Several fully powered randomized trials from abroad show its effectiveness in improving social support of perinatal women (4, 5, 29). It is flexible and easy to implement in low- and middle-income country health facilities by primary healthcare providers in primary healthcare facilities (26) and helps overcome attendance barriers. The present study evaluated the effect of antenatal group-based psychoeducation on enhancing social support for postnatal mothers in primary healthcare settings. A cluster randomized controlled trial was employed to accomplish this. A component of this inquiry was already functional at the facility level, enabling the management of information contamination issues.

## Materials and methods

### Study design and period

We adhered to the CONSORT reporting requirements in documenting our two-arm, parallel-group, single-blind cluster randomized controlled trial, designed to evaluate the impact of a antenatal group-based psychoeducation intervention on improving the social support of postnatal mothers. The study was conducted from 28 March to 1 December 2022 (Supplementary Table S1).

### Setting

The study aimed to implement the intervention across the entire maternal healthcare service within the institution, using clusters (health centers) in Jimma, Ethiopia, as the unit of randomization. In the zone, there are 122 health centers. Primary healthcare units, primarily staffed by midwives and nurses from health centers, typically provide maternal healthcare services to women. Females make up 1,735,628 of the total population in the Jimma Zone (30). The population is characterized by unequal gender norms and limited decision-making capacity. Furthermore, women perform nearly all household tasks, including cooking, caring for the family, and cleaning (31).

### Inclusion and exclusion criteria

Eligibility screening was conducted at both the cluster and individual participant levels. Initially, we identified 32 non-adjusted health centers (clusters) that exhibited comparable characteristics. Those who delivered their maternal healthcare services were female midwives and nurses with at least 6 months of professional experience and were familiar with the local languages and cultures. The study included all pregnant women who were 12–20 weeks gestation, had a PHQ-9 <10 level depression, and were receiving prenatal care at Jimma Zone primary healthcare services (health centers). The study excluded pregnant women who were receiving treatment for a previous mental illness, had hearing impairment, or suffered from severe illness at the time of the trial [for more details, see (32) (p.3)].

### Intervention activities

Midwives or nurses from the study site administered the interventions to pregnant women. Therefore, we provided ample training for midwives or nurses in the maternal healthcare service in the facility. From each intervention cluster, one midwife and/or nurse participated. Pre and post-tests were given for providers to assess their gaps in maternal mental health. Then a detailed group-based psychoeducation training, including PPD screening, was given to midwives and nurses by experienced psychiatrist nurses (assistant professors) and investigators (MSc in clinical midwifery, a PhD fellow in reproductive health at the population and facility health department, certified for positive family therapy). Providers were recruited from their study sites for resource management, convenience of the intervention process, and sustainability of the project. The participants were recruited based on their sex, experience (≥6 months), and communication ability in using the local languages and familiarity with the local culture. Female providers were selected purposely because mothers can discuss their problems and concerns without fear or social stigma with female healthcare providers.

The intervention package given to the provider included relevant aspects of the intervention, such as the antenatal psychosocial assessment tool (PHQ-9), and a procedural guideline for effectively facilitating group psychoeducation. All the antenatal group-based psychoeducation sessions given to the study participants/mothers and procedures for referring women with moderate to severe depression to a nearby hospital were also included. Moreover, methods of monitoring the intervention to decrease the dropout rate of mothers from the intervention and motivating mothers to follow the sessions strictly up to the end-line data collection time were also highlighted for healthcare providers.

The interventions for pregnant women were delivered in groups, with 8–10 pregnant women participating in each group. Over a period of 5 weeks, lasting between 60 and 90 min, baby blues, postnatal depression, PPD symptom recognition, PPD prevention strategies, and the enhancement of social support were addressed. Finally, we extended an invitation to mothers to bring a companion or family member during the final week. Subsequently, they engage in discussions regarding prevalent psychosocial issues that may develop during pregnancy and childbirth, underscoring the importance of family support in the prevention of postpartum depression and stress control. The intervention was exclusively for the treatment groups. Participants in the control group received solely standard prenatal care. A comprehensive description of the intervention is provided in a previous study (32) (p.4–7). Furthermore, Supplementary File S1 contains the details.

### Outcome measurement

The primary outcome variable was social support, which was assessed at 6 weeks postpartum. We also separately assessed other psychosocial variables as a secondary outcome variable to examine their direct effect on the mothers’ overall social support.

### Sample-size determination

Using the following assumptions, we calculated the sample size based on the recommendations for cluster randomized controlled trials (RCTs) for a fixed number of clusters (33, 34). The trial was part of a large study that included three outcome variables (postnatal depression, postnatal depression literacy, and social support). We computed the sample size for each of the three outcome variables and selected the largest sample size based on the following assumptions. The proportion of postnatal depression is 21.9% (9) with a 12% (effect size 0.12) reduction in PPD, 32 clusters accessible (16 clusters in each arm), with a 95% CI and 80% power, and an intra-cluster correlation coefficient of 0.03558 (35). The normal approximation, G-power, a two-sample proportion comparison, and the design effect [DE = 1 + p(m − 1) = 1.7] were employed. Additionally, by adjusting the power, non-response rate, and predetermined number of clusters, the average cluster size for cluster randomization was 20, yielding a total sample size of 640 pregnant women (320 per group). Details can be found in the published paper (32) (p.7).

### Randomization, blinding, and sampling procedure

The randomization unit was the health center. Random sequences generated by SPSS were utilized to allocate health centers in a 1:1 ratio to the intervention and control groups. An individual uninvolved in the study and unaware of the research groups randomized and allocated the clusters into two arms. Furthermore, the study was single blind only the outcome assessors being blinded. They were not informed about the allocation, did not reside in a cluster, and were not trial implementers. Moreover, they were unaware of the intervention's purpose and the objective it aimed to accomplish. Consent was obtained from both cluster and individual participants; a systematic sampling procedure was employed to select the participants. The identification and consent of pregnant women for the baseline survey were conducted prior to the randomization process, hence aiding researchers in mitigating identification biases (32) (p.4).

### Procedure for data collection and quality assurance

We employed a carefully validated, pre-tested, and structured instrument to collect the data. The standard instrument and literature served as the basis for developing the tool. To ensure consistency of meaning, language experts separately translated the English-adapted tool into two local languages, Oromo and Amharic. The training was given to a data collector and supervisor for 2 days by the principal investigator and psychiatrist. Eight midwives, trained for 2 days by the principal investigator and psychiatrist, collected the data under the supervision of two MSc in psychiatry and midwifery professionals. For the pretest, 5% of pregnant women outside the study area underwent the test. Data collection occurred at 12–20 weeks of gestation and 6 weeks postpartum through a face-to-face interview. The data were checked for completeness, quality, and clarity on the spot. All encountered challenges were identified, communicated, deliberated, and resolved prior to the subsequent day for data collecting (32) (p.7).

Furthermore, the validity and reliability of the social support questionnaires were addressed as follows. A functional social support questionnaire was used for the measurement of social support. To adopt a functional social support questionnaire, a cognitive interview was done in the study area among pregnant women (36). And minor change was made to the structural form, “How often?” to “Do you?”; this replaced ordinal-rated response options with dichotomous (i.e., yes/no). Additionally, for this specific study, principal component analysis (PCA) was performed to further validate the item. From 14 items, 1 item (telephone call) was removed which scored less than 5 points. Finally, all the assumptions were checked and fitted. Bartlett's test of sphericity was significant at *P* < 0.000. The Kaiser–Meyer–Olkin (KMO) measure of sampling adequacy was >0.88, and the total variance explained was 66.5%. The reliability of items (inter-item consistency) was checked (Cronbach’s *α* >0.90).

### Statistical analysis

The data analysis was carried out using the SPSS-25 statistical software package. A descriptive analysis was conducted initially to detect missing data, errors, and outliers, facilitating subsequent analysis. To check for missing data, the number of cases missing per variable, the number of variables missing per case, and the pattern of correlations among variables were checked. Based on this analysis, we identified a few missing data points. For those with a few numbers of missed data, imputation techniques were used by revising the original questionnaire and using the average value derived from the other variables’ information to maintain the integrity of the data.

Data from the trial were analyzed using the intention-to-treat analysis procedure. The mean score was analyzed for total and all support resources: those who scored the mean or higher were classified as having “adequate social support,” while those who scored below the mean were classified as having “inadequate social support.” A phi (*φ*) coefficient and relative risk were used to assess effect size between groups (37). Odds ratios were estimated using a multilevel logistic regression model. The presence of cluster-level variability influencing social support was then tested using the intercept-only model and ICC (38). In addition, the measure of variation between clusters was measured using the median odds ratio (MOR) and proportional change in variance (PCV) (39). Finally, the log-likelihood ratio (LR) test was employed to assess model fitness.

Four models were developed during the analysis. Social support was used as the dependent variable. The first model was an empty model employed to assess the impact of cluster variation on social support. The second model was controlled for individual-level variables, the third for community-level variables, and the fourth for both individual- and community-level variables. A bivariate analysis was initially conducted, and factors with *P* < 0.25 were incorporated into the second and third models. Subsequently, variables with a *P* < 0.05 in the second and third models were incorporated into the final model. In the final model, a *P* < 0.05 was employed to establish statistical significance, while the 95% CI was utilized to demonstrate the strength of association and the level of significance, respectively.

## Result

**Figure 1:**
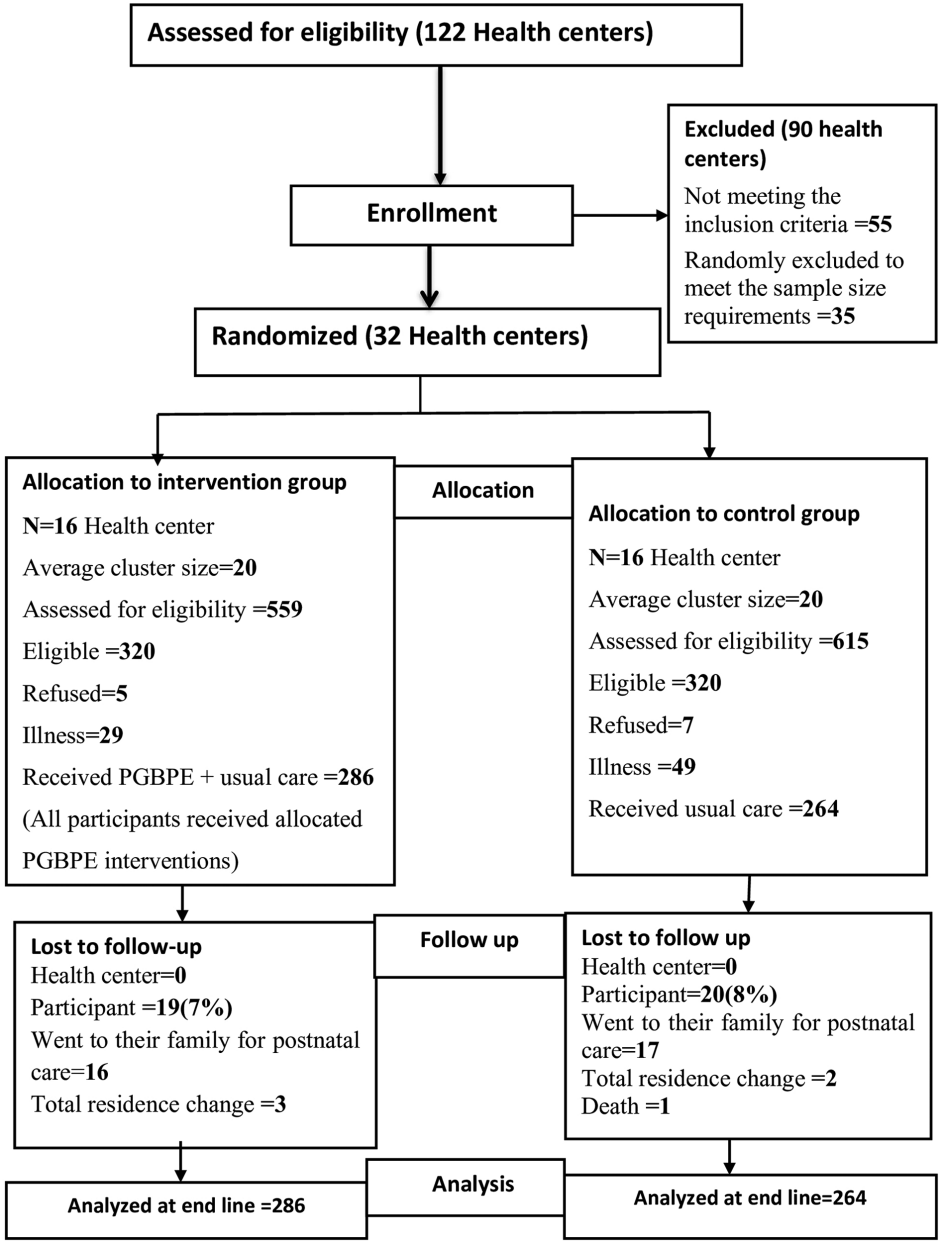
CONSORT 2010 trial flow chart, intervention study, Jimma Zone, 2023.

From 32 non-adjusted health centers (16 each arm), 640 pregnant women's screened for eligibility, and only 550 consented and enrolled in the study, with 286 in the intervention group and 264 in the control group. Furthermore, follow-up resulted in the loss of 19 mothers in the intervention group and 20 mothers in the control group due to a permanent place change, transfer for postnatal care, and death. Finally, end-line data were collected from 511 participants, 267(52.3%) from the intervention group and 244 (47.7%) from the control group. In the end, an intention-to-treat analysis was conducted, and all enrolled mothers were included in the final analysis (*N* = 550) [for more details, see (24)] (Figure 1, trial flow).

The majority of participants, 484 (94.7%) and 521 (71.1%), were married and housewives, respectively. Three hundred thirty-one (60.2%) live in urban areas. More than half (52.9%) were in the age range of 25–34 years old, with a mean age of 27.19 years (SD ± 5.7). Two hundred sixteen participants (39.3%) lack formal education, while 204 (37.1%) have completed primary education. No statistically significant variations were seen between the two groups concerning sociodemographic and obstetric characteristics, with the exception of maternal educational status and parity (Table 1).

**Table 1:** Comparison of background variables (*N* = 550; intervention = 286, controls = 264).

Variable with or without category	Intervention *N* (%)	Control *N* (%)	*P*-value
Residence			
Urban	164 (57.3)	167 (63.3)	0.15
Rural	122 (42.7)	97 (36.7)	
Age			
15–24	86 (30.1)	96 (36.4)	0.37
25–34	159 (55.6)	132((50)	
≥35	41 (14.3)	36 (13.6)	
Marital status			0.54
Married	272 (95.1)	249 (94.3)	
In-relationship	10 (3.5)	9 (3.4)	
Others*	4 (1.5)	6 (2.4)	
Education status			
Informal education	125 (43.7)	91 (34.5)	0.04
Primary (1–8)	104 (36.4)	100 (37.9)	
Secondary (9–12)	35 (12.2)	56 (21.2)	
College/above	22 (7.7)	17 (6.4)	
Job			
Housewives	206 (72)	185 (70.1)	0.67
Private workers	42 (14.7)	44 (16.7)	
Government employees	27 (9.4)	22 (8.3)	
Domestic workers	11 (3.8)	13 (4.9)	
Household income			
<3,000	147 (51.4)	122 (46.2)	0.26
3,001–5,000	76 (26.6)	77 (29.2)	
>5,000	63 (22)	65 (24.6)	
Parity			
Primipara	198 (69.2)	146 (53.3)	0.00
Multipara	88 (30.8)	118 (44.7)	
Unplanned pregnancy for the mother			
Yes	65 (22.7)	58 (22)	0.81
No	221 (77.3)	206 (78)	
Unplanned pregnancy for the father			
Yes	71 (24.8)	59 (22.3)	0.49
No	215 (75.2)	205 (77.7)	
History of mental health			
Yes	14 (4.9)	10 (3.8)	0.56
No	272 (95.1)	254 (96.2)	
Social support			
Adequate	63 (22.03)	60 (22.73)	0.84
Inadequate	223 (77.97)	204 (77.27)	
PPD literacy			
Literate	78 (27.27)	77 (29.17)	0.62
Illiterate	208(72.73)	187 (70.83)	

Others* = divorced, separated, widowed.

**Figure 2:**
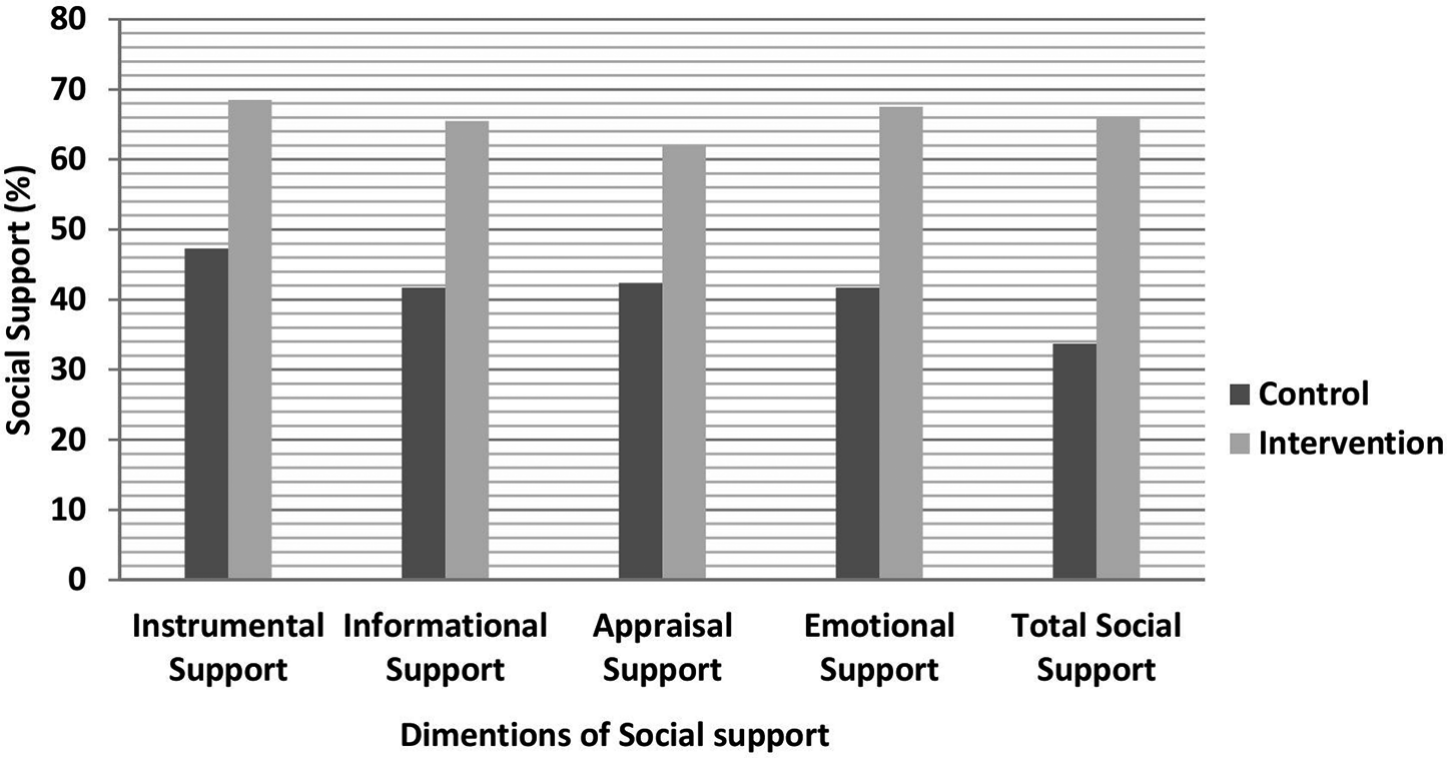
Comparison of all dimensions of social support by intervention and control groups, 2023.

Statistical analysis revealed a substantial difference in all dimensions of social support between the intervention and control groups, with no differences observed at baseline. Mothers in the intervention group received total social support of 66.4%, compared to 33.3% in the control group, with *P* < 0.001 (for more details, see Figure 2).

Furthermore, the phi (*φ*) coefficient, relative risk, and multilevel logistic analysis were conducted for total social support. The phi (*φ*) coefficient = 0.331 with df = 1 indicates a very strong effect size (37). Similarly, mothers who were under the intervention arms and received antenatal group-based psychoeducation were 2.04 times more likely to have postnatal social support (RR = 2.044, 95% CI: 1.684–2.481) compared to those who were under the control arms and received the usual care. Finally, mixed-effects multilevel logistic regression analysis for total social support indicates that after adjusting for individual and community-level variables, the final model shows the intervention increased the total social support by 3.61 (AOR = 3.61, 95% CI: 2.14–6.09). Furthermore, mothers who had emotional support from their partner (AOR = 1.61, 95% CI: 1.00–2.59) and those who had good support from their mother (AOR = 4.25, 95% CI: 1.78–10.15) were more likely to have adequate social support. On the contrary, mothers who had postpartum depression (AOR = 0.06, 95% CI: 0.02–0.19), who used unhealthy ways to deal with stress (AOR = 0.28, 95% CI: 0.17–0.44), and who felt loneliness (AOR = 0.28, 95% CI: 0.11–0.69) were less likely to have adequate social support (Table 2).

**Table 2:** Multilevel logistic regression analysis (*N* = 550).

Variables	Model 2 AOR (95% CI)	Model 3 AOR (95% CI)	Model 4 AOR (95% CI)
Individual-level factors			
Education status			
Informal education	**1**		
Primary (1–8)	1.573 (0.464–5.330)		
Secondary (9–12)	1.386 (0.700–2.741)		
College/above	1.087 (0.655–1.802)		
Job			
Housewife	1		
Merchant	0.555 (0.160–1.924)		
Gov't employee	1.178 (0.400–3.469)		
Domestic worker	1.462 (0.755–2.828)		
Parity			
Primi	1		
Multipara	0.734 (0.452–1.192)		
Unwanted pregnancy (mother)			
Wanted	1		
Unwanted	1.262 (0.727–2.192)		
Complication during labor			
Yes	2.033 (0.888–4.656)		
No			
Loneliness			
Yes	**0.244** (**0.097–0.611)**		**0.278 (0.113–0.685)**
No	**1**		1
Emotional coping			
Yes	0.658 (0.388–1.113)		
No	1		
Problem-based coping			
Yes	1.048 (0.615–1.788)		
No	1		
Dysfunctional coping			
Yes	**0.219** (**0.129–0.370)**		**0.276 (0.172–0.444)**
No	**1**		**1**
Postnatal depression literacy			
Good	1.443 (0.886–2.351)		
Poor	**1**		
Postpartum depression			
Yes	**0.037** (**0.011–0.131)**		**0.057 (0.017–0.194)**
No	**1**		**1**
Community-level variable			
Marital status			
Married		1	
In-relationship		0.684 (0.140–3.346)	
Others*		0.519 (0.149–1.815)	
Group-based psychoeducation			
Yes		**3.898 (2.538–5.988)**	**3.607 (2.136–6.090)**
No		**1**	**1**
Relationship with in-laws			
Good		0.688 (0.451–1.050)	
Poor		**1**	
Partner emotional support			
Good		**2.899 (1.910–4.400)**	**1.610 (1.002–2.588)**
Poor		**1**	**1**
Having support from her mother			
Good		**6.865 (3.158–14.924)**	**4.246 (1.776–10.152)**
Poor		1	
Unwanted pregnancy (by partner)			
Wanted		1	
Unwanted		**0.621 (0.387–0.997)**	

Bold value signifies *P* < .05. Others* = divorced, separated, widowed.

## Discussions

This study's finding indicated that antenatal group-based psychoeducation significantly improved all dimensions of postnatal social support in the intervention group relative to the control group. It confirmed the hypothesis that group-based psychoeducation given during early pregnancy when women ' less likely depressed helps improve the level of social support among postnatal mothers. It is supported by controlled trials and systematic review and meta-analysis study (4, 5, 29) which show the effectiveness of psychoeducation intervention in improving all dimensions of social support of postnatal women.

Psychoeducation works primarily by providing the client with comparable types of problems and their families’ knowledge of the various aspects of the illness and its prevention and therapy. As a result, they can work with professionals and families to achieve a better overall outcome (40, 41). The idea is that the more informed the mothers and informal caregivers are about PPD and the necessity of social support, the greater assistance the mother receives (28, 41, 42). Therefore, in this study, the family was part of the intervention; this helped the mother to be easily understood by family members and get adequate social support to cope with stressful situations and prevent PPD (32). This is supported by a controlled trial of family psychoeducation intervention on social support of postnatal women (4, 5, 43). Furthermore, psychoeducation increases the social competence of the mother by increasing her awareness and understanding of PPD, hence facilitating better social interactions (5, 44).

Moreover, psychoeducation can be administered individually or in groups to clients with similar types of problems and their families (40, 41). Similarly, in this study, all pregnant women are at risk of perinatal depression, and almost all risk factors are common for all mothers, caused by hormonal changes related to pregnancy and the demands of childcare (45). In a similar vein, in this study, the intervention was administered in groups, which has its own set of advantages for the mothers. The groups primarily were support groups for each other, addressing the common questions asked by mothers through discussion and experience sharing, enabling them to understand the significance of social support during this period and to share their own experiences of obtaining it (43, 46, 47).

Moreover, in Ethiopia, strong social support networks, predominantly centered on extended family and community connections, illustrate a collectivist cultural perspective, as individuals rely significantly on relatives and neighbors for emotional, practical, and financial assistance (48, 49). Evidence shows that collectivist culture significantly impacts the characteristics and availability of social support (48). WHO indicates that postpartum women, which involves seclusion and the provision of heightened care to mothers and neonates in the first 40 days postpartum, were protective practices that involved direct interpersonal care (50). In a similar vein, culturally, it is considered taboo in Ethiopia to abandon postpartum mothers for the first 40 days after delivery. Similar to this study, the statistical analysis revealed that mothers with supportive partners and mothers who had support from their mothers were more likely to have adequate social support compared to their counterparts.

However, collectivist culture is sometimes affected by cultural norms that can influence how individuals seek and receive support; sometimes it prioritizes the needs of the collective over individual needs, affects women's decision-making capacity in the community, and causes social stigma, such as in the case of mental illness (48, 51). Similarly, the current study revealed that mothers who had postpartum depression, feelings of loneliness, and ineffective coping had lower social support compared with their counterparts. One primary explanation for this phenomenon is that mothers experiencing PPD, dysfunctional coping mechanisms, and feelings of isolation often choose to withdraw themselves from social interactions. These factors cause irritation, a desire for solitude, and other associated emotions such as perceived stigma on the mothers (3). In the future, it is critical to take into account the three factors outlined above when promoting social support.

Generally, this research offered a way for midwives and nurses to enhance social support at the primary maternal healthcare level. Additionally, these results will offer new insights into the body of knowledge and assist policymakers and other interested bodies in preparing pertinent resources, advancing their maternal healthcare initiatives, and enhancing the social support status of mothers. The study's results are equally significant for other researchers seeking to pursue other investigations. It is recommended that midwives and other healthcare professionals in the primary care system be familiarized with and taught about this intervention technique.

## Limitations

The trial was part of a large study that included three outcome variables (postnatal depression, postnatal depression literacy, and social support). This trial presents specific limitations. Mothers experiencing moderate to severe depression were excluded from the trial, and the intervention's effects were not evaluated during pregnancy; outcomes were compared solely using end-line data. We had both ethical and methodological limitations for this. The first one was that we encountered a serious ethical issue: there is no intervention tailored to maternal mental health in our clinical setup, so we could not leave those mothers in the control group without care after we knew they were depressed. To address this concern, we recruited mothers between 12 and 20 weeks of gestation, when depression is less likely; however, a few depressive mothers were excluded from both the intervention and control groups and referred to nearby hospitals’ psychiatric outpatient departments. However, we can still generalize this finding to pregnant women 12–20 weeks of gestation with normal and mild depressive symptoms who attend ANC in primary healthcare facilities. Furthermore, we compared the outcome variable only based on end-line data because pregnant and postnatal mothers have different experiences, so we used the baseline data to assess the background information difference (32).

## Conclusions and recommendation

This study demonstrated that antenatal group-based psychoeducation provided by primary healthcare practitioners effectively enhanced all dimensions of social support. Moreover, family involvement was essential for all outcomes produced by the intervention. The incorporation of this intervention into the maternal healthcare system can enhance both the physical and emotional well-being of women and foster maternal mental health. Consequently, this experiment warrants consideration for wider adoption, in accordance with the Ministry of Health's initiatives to enhance mental health within primary healthcare settings.

## Data Availability

The original contributions presented in the study are included in the article/Supplementary Material, further inquiries can be directed to the corresponding author.
